# A Relook into the Flavonoid Chemical Space of *Moringa oleifera* Lam. Leaves through a Combination of LC-MS and Molecular Networking

**DOI:** 10.1155/2023/1327886

**Published:** 2023-09-25

**Authors:** Dakalo Lorraine Ndou, Ashwell Rungano Ndhlala, Nikita Tawanda Tavengwa, Ntakadzeni Edwin Madala

**Affiliations:** ^1^Department of Chemistry, Faculty of Science, Engineering and Agriculture, University of Venda, Private Bag X5050, Thohoyandou 0950, South Africa; ^2^Green Biotechnologies Research Centre of Excellence, Department of Plant Production, Soil Science and Agricultural Engineering, University of Limpopo, Private Bag X1106, Sovenga 0727, South Africa; ^3^Department of Biochemistry and Microbiology, Faculty of Science, Engineering and Agriculture, University of Venda, Private Bag X5050, Thohoyandou 0950, South Africa

## Abstract

*Moringa oleifera* Lam. is a functional tree that is known to produce a variety of metabolites with purported pharmacological activities. It is frequently called the “miracle tree” due to its utilization in numerous nutraceutical and pharmacological contexts. This study was aimed at studying the chemical space of *M. oleifera* leaf extracts through molecular networking (MN), a tool that identifies metabolites by classifying them based on their MS-based fragmentation pattern similarities and signals. In this case, a special emphasis was placed on the flavonoid composition. The MN unraveled different molecular families such as flavonoids, carboxylic acids and derivatives, lignin glycosides, fatty acyls, and macrolactams that are found within the plant. In silico annotation tools such as network annotation propagation (NAP) and DEREPLICATOR, an unsupervised substructure identification tool (MS2LDA), and MolNet enhancer were also explored to further compliment the classic molecular networking output within the Global Natural Product Social (GNPS) site. In this study, common flavonoids found within *Moringa oleifera* were further annotated using MS2LDA. Utilizing computational tools allowed for the discovery of a wide range of structurally diverse flavonoid molecules within *M. oleifera* leaf extracts. The expansion of the flavonoid chemical repertoire in this plant arises from intricate glycosylation modifications, leading to the creation of structural isomers that manifest as isobaric ions during mass spectrometry (MS) analyses.

## 1. Introduction

*Moringa oleifera* Lam. has been reported to have a broad range of pharmacological activities such as antimicrobial, anti-inflammatory, hypotensive, antidepressant, antioxidant, antidiabetic, hypoglycemic, and immunomodulatory properties [1–3]. The chemical constituents of the stems, leaves, flowers, pods, and seeds of *M. oleifera* have been analyzed to determine the presence of bioactive compounds, and they were found to contain various secondary metabolites such as phenolic acids, sterols, terpenoids, flavonoids, alkaloids, and sugars and anticancerous agents such as glucosinolates, isothiocyanates, glycoside compounds, and glycerol-1-9-octadecanoate which have nutritional, pharmaceutical, and antimicrobial properties [4–8]. However, studies on this plant have shown that the presence of the bioactive compounds is dependent on various factors such as the geographical origin, the harvesting season, and cultivation conditions [9].

Metabolomics is a field of study that gives a systematic view of the unique chemical fingerprints of metabolites and their small changes in a specific cellular process [10]. A metabolomics study includes sample preparation, analytical measurement, data analyses, and interpretation [11–13]. Mass spectrometry (MS) and nuclear magnetic resonance (NMR) techniques are reported to be the analytical workhorses of metabolomics [14–16]. A molecular family (MF) is constructed by the grouping of structurally related molecules that generate similar fragmentation patterns. To do this on a larger scale, computational tools such as molecular networking (MN) have been developed [12, 17–19]. MN is a popular tool in the analysis of tandem MS- (MS/MS-) based metabolomics data. MN is fundamentally based on the observation that two structurally related molecules share fragment ion patterns when subjected to MS/MS and aid the elucidation of the structure/identity of many compounds of untargeted MS [20–22]. MN has led to the development of the Global Natural Product Social (GNPS) which is a molecular networking and data-sharing web-based platform [23, 24].

GNPS is widely used by scientists from various platforms in the fields of chemistry, microbiology, forensics, and many more to perform sample classification with the objective to give identity of the content thereof. GNPS facilitates data, stores knowledge, enables sharing, and promotes reproducible data analysis [21]. GNPS can be used for molecular networking and is currently the only public infrastructure that enables molecular networking [23]. The related molecules as depicted in MN can be viewed online at GNPS or on Cytoscape for analysis [13, 25]. Other tools in GNPS include network annotation propagation (NAP) and DEREPLICATOR and an unsupervised substructure identification tool called MS2LDA, all of which are meant to strengthen metabolite identification through MN. These tools are used to complement classic MN output and integration using MolNetEnhancer within GNPS [26].

In this study, the chemical space of *M. oleifera* was studied through computational tools within GNPS. Molecular networking was used to reveal the molecular families of this plant, and the unsupervised substructure annotation tool (MS2LDA) was used to annotate the Mass2Motifs of some of the flavonoids that are found within *M. oleifera* by depicting similar fragmentations and neutral losses.

## 2. Materials and Methods

### 2.1. Chemicals and Reagents

Methanol (99% CP) was purchased from Associated Chemical Enterprises (Johannesburg, South Africa). Ultrapure water using a Direct-Q 5UV distiller (Massachusetts, the United States of America) was used for the preparation of the 80% methanol solution. The extraction was performed on a DIAB MX-RL-Pro dragon shaker. Chromatographic separation of the metabolites in the extracts was done using a reverse phase Shim-pack Velox C18, 2.1 × 100 mm, 2.7 *μ*m (Columbia, USA). The UPLC was connected to a Shimadzu 9030 LC, qTOF-MS detector (Shimadzu, Kyoto). The solvents used for the chromatographic runs were methanol and formic acid, which were purchased from ROMIL Pure Chemistry (Cambridge, UK).

### 2.2. Plant Collection and Sampling

Leaves were collected from cultivated *M. oleifera* plants in multiple households across various villages within the Vhembe District of the Limpopo Province of South Africa. After being harvested, these leaves were kept in darkness while being transported to the University of Venda. Subsequently, the leaves were air-dried in the absence of light at room temperature and then finely ground into a powder using a blender. This powdered form was stored in a dark environment until the metabolite extraction process.

### 2.3. Preparation of the Extract

A modified version of a previously described extraction method [27] was utilized. In summary, 1 gram of ground leaf powder from each cultivar was mixed with 10 mL of 80% aqueous methanol (MeOH) and shaken overnight using a dragon shaker. The resulting mixture was then centrifuged at a high speed of 5000 × *g* for 20 minutes at a temperature of 25°C. The supernatant liquid was transferred into an Eppendorf tube, filtered through 0.22 *µ*m filters into a vial, and subjected to UPLC-qTOF-MS analysis. Any remaining supernatant solutions were stored in a refrigerator.

### 2.4. Ultrahigh Performance Liquid Chromatography-Quadruple Time-of-Flight Mass Spectrometry (UHPLC-qTOF-MS)

To analyze the extracts, the LCMS-9030 qTOF instrument from Shimadzu Corporation in Kyoto, Japan, was employed, following the method outlined by Ramabulana et al. in 2021 [26]. Liquid chromatography separation took place on a Shim-pack Velox C18 column (100 mm × 2.1 mm, particle size 2.7 *µ*m) housed in a column oven maintained at 55°C. A binary mobile phase gradient consisting of solvent A (0.1% formic acid in Milli-Q water) and solvent B (methanol with 0.1% formic acid) was used. An injection volume of 3 *µ*L was applied to all samples. The gradient conditions were as follows: 10% B for 3 minutes, 10–60% B over 3–40 minutes, 60% B from 40 to 43 minutes, and 90% B from 43 to 45 minutes (maintained for 3 minutes), returning to initial conditions from 48 to 50 minutes, followed by a 3-minute column re-equilibration time. The chromatographic effluents were analyzed using a qTOF high-definition mass spectrometer in a negative electrospray ionization mode. The instrument was calibrated with sodium iodide (NaI), and both MS1 and MS2 data were simultaneously generated through a data-dependent acquisition (DDA) mode for all ions within an *m*/*z* range of 100–1000 and an intensity threshold of 5000. Fragmentation experiments were conducted using argon as a collision gas, with collision energy of 30 eV and a spread of 5 eV. The MS settings were as follows: interface voltage of −4.0 kV, interface temperature of 300°C, nebulization and dry gas flow rate of 3 L/min, heat block temperature of 400°C, DL temperature of 280°C, and detector voltage of 1.8 kV.

### 2.5. Molecular Networking and Metabolite Annotation

The creation of a molecular network was performed using the GNPS website (https://gnps.ucsd.edu) through an online workflow (https://ccms-ucsd.github.io/GNPSDocumentation/), accessed on August 17, 2021. The data underwent filtering by removing MS/MS fragment ions within ±17 Da of the precursor *m*/*z* and selecting only the top 6 fragment ions in the ±50 Da window across the spectrum. The precursor ion mass tolerance was set at 2.0 Da, and a MS/MS fragment ion tolerance of 0.5 Da was applied. The resulting network was filtered to have a cosine score above 0.7 and more than 6 matched peaks for the edges, while nodes were connected if they appeared in each other's respective top 10 most similar nodes. Molecular families were limited to a size of 100, and low-scoring edges were eliminated until the size was below this threshold. The network spectra were then searched against GNPS' spectral libraries using the same filtering criteria. Finally, the visualization of the molecular network was carried out using Cytoscape software. Empirical formulae were generated from accurate mass and fragmentation patterns obtained from the MS2 data to annotate all matched nodes and some unmatched nodes of metabolites. These annotations were compared to dereplication databases such as the KNApSAcK chemical database. Substructure annotation was achieved using MS2LDA through the ms2lda.org web interface within GNPS. Structural searches were performed according to the protocol recently outlined by Moyo et al. [28].

## 3. Results and Discussion

**Figure 1:**
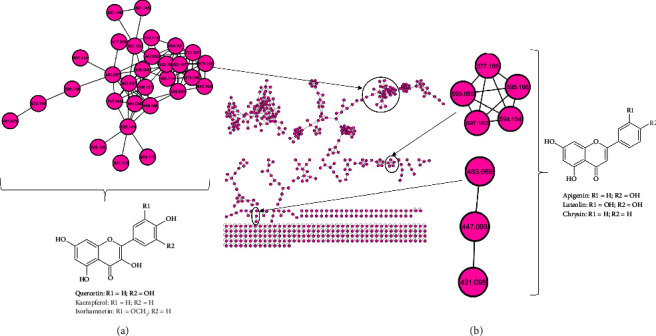
Molecular network of *Moringa oleifera* Lam. leaf extracts as analyzed by liquid chromatography-tandem mass spectrometry using electrospray ionization in negative mode (center), with two different kinds of flavonoids highlighted: (a) flavonols and (b) flavones.

MS/MS spectra of six methanolic extracts from the *M. oleifera* cultivars were compared to find similarities in the fragmentation patterns (i.e., same fragment ions or similar neutral losses) of the metabolites. Metabolites that are structurally related and have similar gas phase chemistries were grouped into molecular families based on a cosine score ≥0.7 [26]. Using molecular networking, the MS/MS spectra were organized into 565 nodes, with 338 clustered into 38 different molecular families (with a minimum of two nodes connected by an edge) based on GNPS spectral matching. A total of 227 nodes were not clustered into a molecular family and were represented as individual nodes at the bottom of the network (Figure 1).

Previous studies have shown the presence of structurally diverse flavonoid molecules in the plant extracts. However, most of the work conducted in this study was through classical means of chemical identification where obtained mass spectrometry (MS) signals were compared with what is already known in the literature. This approach, however, has negative connotation owing to the limitation on information of some uncharacterized metabolites. A molecular network is a computational method aimed at metabolite identification by classifying metabolites based on their MS-based fragmentation pattern similarities and signals.

### 3.1. Exploration of the Chemical Space of *Moringa oleifera*

**Figure 2:**
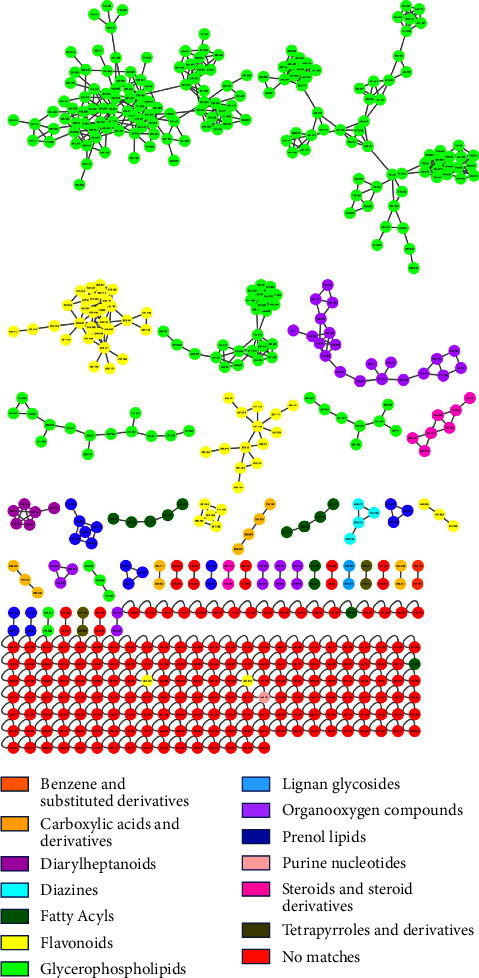
An enhanced molecular network in which nodes are highlighted based on their chemical superclass based on MS2LDA, network annotation propagation (NAP), and DEREPLICATOR outputs.

*Moringa oleifera* is well known for its nutraceutical and pharmacological metabolic profiles which are characterized by the presence of flavonoids, glucosinolates, and chlorogenic acids. In this study, the metabolic profile of *M. oleifera* was studied with the help of molecular networking from the GNPS website. MolNetEnhancer (Figure 2) represents the metabolomes of this plant that were observed in this study. The node annotations of MolNetEnhancer were based on MS2LDA, network annotation propagation (NAP), and DEREPLICATOR outputs. It was observed that this plant contains 16 different classes of metabolites including carboxylic acids and derivatives, fatty acyls, flavonoids, glycerophospholipids, lignin glycosides, macrolactams, macrolides, naphthacenes, organooxygen compounds, prenol lipids, purine nucleotides, and tetrapyrroles and derivatives. A study by the authors in [29] revealed the presence of hydroxyl fatty acids, phenolic acids, flavonoids, intact glucosinolates, sulfolipids, and phenolic acid derivatives' metabolite classes.

Flavonoids have been reported to be the predominant group of metabolites in *M. oleifera* leaf extracts with kaempferol and quercetin derivatives being the most predominant group [30]. Flavonoids are naturally occurring polyphenols that accumulate in the edible parts of plants, more particular in fruits and vegetables [31]. Flavonoids can further be subdivided into flavones, flavanols, flavanones, flavonols, flavanonols, isoflavones, and anthocyanins. In this study, much attention was given to flavones and flavonols. Flavones and flavonols have antioxidant effects and are essential for protecting plants from UV radiation [32]. Quercetin and kaempferol (Figure 1(a)), among others, are abundant dietary flavonols found in fruits and vegetables. Flavonols have various health benefits which include cardiovascular and antioxidant properties. Luteolin and apigenin (Figure 1(b)) are the main flavones that are found in fruits and vegetables and have a wide range of biological effects such as anticancer, antioxidant, and anti-inflammation properties [33–35].

In this study, a total of 52 flavonoids were detected. Kaempferol derivatives are known to have a major fragment ion at *m*/*z* 285, and quercetin derivatives are known to have a major fragment ion at *m*/*z* 301, both indicating the aglycone moiety thereof. Another common flavonoid in *M. oleifera* leaves is isorhamnetin, and derivatives of this flavonoid have a major fragment ion at *m/z* 314, again indicating the aglycone moiety. The detailed mass information of selected flavonoids that were annotated in this study is shown in Table 1.

**Figure 3:**
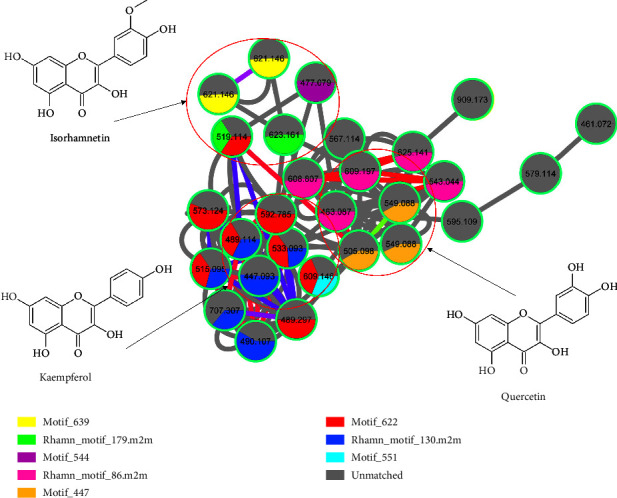
Metabolite annotation using MolNetEnhancer by MS2LDA where the colored parts represent the flavonoids that make up the Mass2Motif.

There are other various tools that are available in GNPS that compliment molecular networking. Such tools are *in silico* metabolite annotation tools such as network annotation propagation (NAP) and dereplication. These tools perform *in silico* fragmentation of known structures and then search against chemical databases. Within the GNPS, there is another valuable resource known as mass spectrometry-mass spectrometry latent Dirichlet allocation (MS2LDA). MS2LDA is an unsupervised computational technique that reveals inherent substructures within compounds by analyzing intricate mass spectrometry (MS) data. This algorithm operates on an unsupervised basis, automatically detecting patterns and substructures within the complex MS data. This capability allows for the identification of shared substructures or fragmentation patterns among compounds. MS2LDA decomposes each molecule into one or more Mass2Motifs which allow for more efficient molecular grouping, searching, and exploration [36]. Mass2Motifs consist of similar fragments and neutral losses [37, 38]. The structural annotation of the Mass2Motifs is straightforward and less complex because Mass2Motifs represent smaller substructures [39].  Figure 3 represents the metabolite annotation using MolNetEnhancer and by MS2LDA of flavonoids found in *M. oleifera* leaves. The colored parts are representative of the flavonoids that make up the Mass2Motifs. Quercetin, kaempferol, and isorhamnetin are the major flavonols that are represented in Figure 3. It is observed that some of these flavonols share the same Mass2Motif owing to their similar fragments and neutral losses. For example, the flavonoids with precursor ion (M-H)^−^ at *m*/*z* 533.088 and at *m*/*z* 592.785 share the same Mass2Motif because they share similar fragments due to the similar aglycone structure.

### 3.2. Quercetin Flavonoids

**Figure 4:**
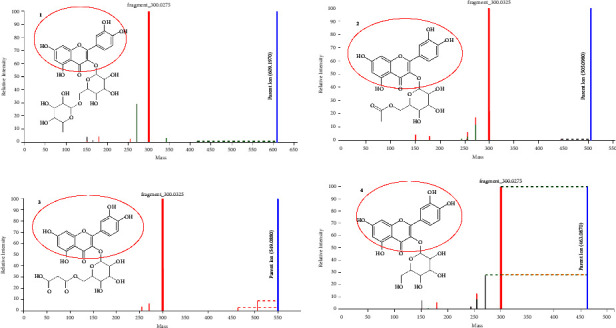
Fragmentation spectra of some quercetin-related flavonoids as annotated by MS2LDA.

Quercetin is a flavonoid that is abundantly found in fruits and vegetables and can be used as a nutritional supplement. This compound has been reported to prevent diseases such as tumors, lung and cardiovascular diseases, and some forms of cancer [40–42].  Figure 4 shows the fragmentation spectra of four quercetin-related flavonoids as annotated by rhamn_motif_86.m2m and motif_447 mass2motifs on MS2LDA approach. Rhamn_motif_86.m2m (a quercetin-related motif) indicated the presence of a quercetin aglycone with diagnostic fragments at *m*/*z* 301, 300, 255, and 179 and a neutral loss of 106 amu. Motif_447 also indicated the presence of a quercetin aglycone with fragments at *m*/*z* 301, 300, 271, and 255 and neutral loss of 44 amu. Quercetin flavonoids are characterized by a deprotonated quercetin aglycone fragment at *m*/*z* 300/301, and other characteristic product ions of *m*/*z* 271, 255, 179, and 151 further confirm the identity of the quercetin aglycone [43]. Compound **1** gave a precursor ion (M-H)^−^ at *m*/*z* 609.197 and a fragmentation ion at *m*/*z* 300.028 due to the loss of the rhamnose and glucose sugars was seen as a base peak. Therefore, this compound was identified as quercetin rutinoside [44]. Compound **2** gave a precursor ion (M-H)^−^ at *m*/*z* 505.098 and showed a fragment ion at *m*/*z* 445.078 due to the loss of the acetyl moiety (60 amu) and a further loss of the hexosyl moiety (162 amu) resulting in the fragment at *m*/*z* 300. This compound was thus identified as quercetin acetyl hexose [45]. Compound **3**, which was identified as quercetin malonyl hexose, gave a precursor ion (M-H)^−^ at *m*/*z* 549.089 showing a fragment at *m*/*z* 505 due to the loss of an acetyl (44 amu) and another fragment at *m*/*z* 463 due to the loss of the malonyl moiety (86 amu). A further loss of the hexosyl moiety (162 amu) led to the fragment ion at *m*/*z* 300 [46]. Compounds **2** and **3** share the same Mass2Motif due to the similar neutral losses which are incurred due to the loss of the hexose moiety. Compound **4** gave a precursor ion (M-H)^−^ at *m*/*z* 463.087 and a fragmentation ion at *m*/*z* 300.028 due to the loss of hexose. This compound was identified as quercetin hexose [47].

### 3.3. Kaempferol Flavonoids

**Figure 5:**
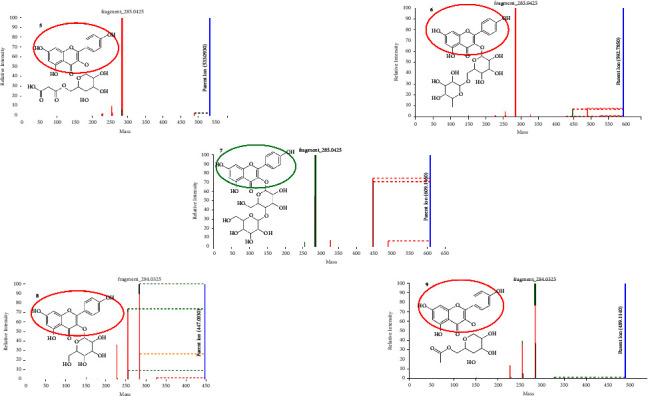
Fragmentation spectra of some kaempferol-related flavonoids in *M. oleifera* as annotated by MS2LDA.

Kaempferol is a flavonoid that is found in various plant parts such as seeds, leaves, fruits, flowers, and even vegetables. It has been referred to as a nutraceutical, owing to its medicinal and nutritional benefits [48]. For instance, kaempferol and its glycosides have been reported to have cardioprotective, neuroprotective, anti-inflammatory, antioxidant, and anticancer activities [49, 50].  Figure 5 shows the fragmentation spectra of five different kaempferol-related flavonoids as annotated by rhamn_motif_130.m2m (kaempferol-related motif) and motif_622, and motif_551 mass2motifs on MS2LDA approach. Rhamn_motif_130.m2m is a characteristic of kaempferol with diagnostic fragments at *m*/*z* 285, 284, 255, and 227. Motif_622 also represents kaempferol with diagnostic fragments at *m*/*z* 285, 284, and 255 and a neutral loss of 68 amu. Motif_551 was characterized by diagnostic fragments at *m*/*z* 283 and 110 and neutral losses of 162, 167, 182, 193, and 194 amu. Kaempferol flavonoids are characterized by a deprotonated kaempferol aglycone fragment at *m*/*z* 284/285, and other characteristic product ions at *m*/*z* 255 and 227 further confirm the identification of the kaempferol aglycone [43]. Compound **5** gave a (M-H)^−^ ion at *m*/*z* 533.093 while its MS/MS fragmentation gave a base peak at *m*/*z* 285.043, due to the loss of the malonyl hexose moiety, and was thus identified as kaempferol malonyl hexose [51]. Compound **6** gave a (M-H)^−^ ion at *m*/*z* 592.785, while its MS/MS fragmentation gave a base peak at *m*/*z* 285.043 due to the loss of the rutinoside sugar and was identified as kaempferol rutinoside [52]. Compound **7** was identified as kaempferol diglucoside with a precursor ion at *m*/*z* 609.146 (M-H)^−^ with a fragmentation peak at *m*/*z* 285.043. This compound also has fragments at *m*/*z* 446.089 and 447.098 due to the loss of the two hexose moieties (162 + 162 amu) [53]. Compound **8**, which was identified as kaempferol hexose, has a precursor ion (M-H)^−^ at *m*/*z* 447.093 with a fragmentation ion at *m*/*z* 284.033 which is due to the loss of the hexose sugar (162 amu) [54]. Compound **9** gave a precursor ion (M-H)^−^ at *m*/*z* 489.114 with a fragmentation ion at *m*/*z* 284.033 due to the loss of an acetyl hexose moiety. This compound was thus identified as kaempferol acetyl hexose [30]. Compounds **5** and **6** were annotated by motif_622, compound **7** was annotated by motif_551, and compounds **8** and **9** were annotated by rhamn_motif_130.m2m, as shown in Figure 3.

### 3.4. Isorhamnetin flavonoids

**Figure 6:**
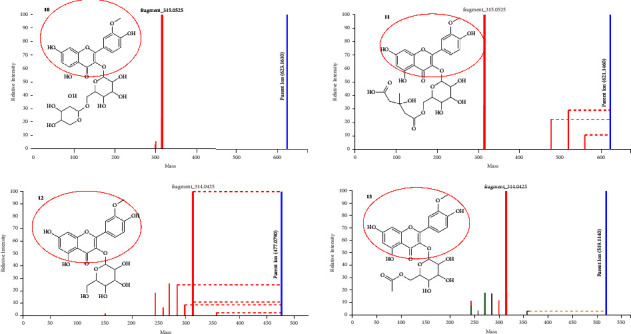
Fragmentation spectra of some isorhamnetin-related flavonoids in *M. oleifera* as annotated by MS2LDA.

Isorhamnetin is commonly present in the leaves, flowers, and fruits of many plants and also forms part of a daily diet. This flavonoid hosts various pharmacological properties such as cardiovascular protection, antibacterial, antiviral, antioxidation, anti-inflammation, and antitumor properties [55–57].  Figure 6 shows the fragmentation spectra of 4 isorhamnetin-related flavonoids as annotated by rhamn_motif_179.m2m (rhamnetin (=7-methylquercetin) motif) and motif_639, and motif_544 mass2motifs on MS2LDA approach. Rhamn_motif_179.m2m represents 7-methylquercetin with diagnostic fragments at *m*/*z* 315, 314, 300, and 299 and a neutral loss of 32 amu. Motif_639 represents isorhamnetin with diagnostic fragments at *m*/*z* 559, 519, 477, 315, and 314 and neutral losses of 62, 102, and 144 amu. Motif_544 also represents isorhamnetin with diagnostic fragment ions at *m*/*z* 315, 314, 299, 285, 271, 257, and 243 with neutral losses of 162, 163, 178, and 192 amu. Isorhamnetin flavonoids are characterized by a deprotonated isorhamnetin aglycone fragment at *m*/*z* 314/315, and other characteristic product ions at *m*/*z* 300, 271, 255, and 227 further confirm the identification of the isorhamnetin aglycone [43]. Compound **10** gave a precursor ion (M-H)^−^ at *m*/*z* 623.161 with a fragmentation ion at *m*/*z* 315.053 indicating the loss of a rutinoside sugar. This compound was thus identified as isorhamnetin rutinoside [58]. Compound **11**, which was identified as isorhamnetin hydroxymethylglutaroyl hexose, gave a precursor ion (M-H)^−^ at *m*/*z* 621.146 and a fragmentation ion at *m*/*z* 315.053. This compound also gave fragments at *m*/*z* 559 and *m*/*z* 519.118 which were due to the loss of hexosy and hydroxymethylglutaroyl moieties [6]. Another fragment was observed at *m*/*z* 477.103 which was due to the loss of the hydroxymethylglutaroyl moiety (144 amu). Compound **12** gave a precursor ion (M-H)^−^ at *m*/*z* 477.079 with a fragmentation ion at *m*/*z* 314.043 due to the loss of the hexose sugar (162 amu). This compound was thus identified as isorhamnetin hexose [59]. Compound **13** gave a precursor ion (M-H)^−^ at *m*/*z* 519.114 and a fragmentation ion at *m*/*z* 314.043 due to the loss of the acetyl hexose moiety. This compound was identified as isorhamnetin acetyl hexose (204 amu) [60].

### 3.5. Apigenin, Luteolin, and Chrysin Flavonoids

Apigenin is a natural flavonoid found in a daily diet and has gained attention due to its low toxicity and various nutritional and biological properties. Because of the medicinal and nutritional properties, it is thus termed a nutraceutical. This flavonoid has antioxidant, antimicrobial, anti-inflammatory, and anticarcinogenic properties [61–63]. Chrysin is also a natural flavonoid that is found in many plants and bee products. This flavonoid has been reported to have a variety of biological properties such as anti-inflammation, antioxidation, anticancer, antibacterial, antidiabetic, and neuroprotective effects [64–66]. Luteolin is a flavonoid that is found in medicinal plants, fruits, and vegetables. Plants that are rich in this flavonoid are often used for the treatment of various diseases such as inflammatory disorders, hypertension, and cancer [67, 68].

**Figure 7:**
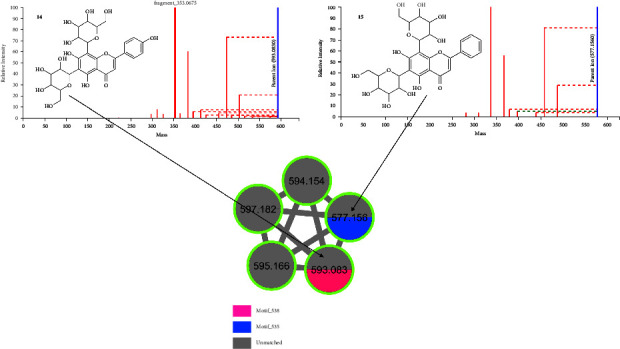
Fragmentation spectra of apigenin-6,8-C-dihexose (**14**) and chrysin-6,8-C-diglucoside (**15**) in *M. oleifera* as annotated by MS2LDA.

Figure 7 shows the fragmentation spectra of an apigenin-related flavonoid and a chrysin-related flavonoid as annotated by motif_535 and motif_538 mass2motifs on MS2LDA approach. Motif_538 is characterized by fragment ions 503, 473, 413, 395, 383, and 353 and neutral losses of 90, 120, 180, and 198 amu. Motif_535 is characterized by fragments at *m*/*z* 337, 367, 379, 457, and 497 and neutral losses of 90, 120, and 198 amu. Compound **14** gave a precursor ion (M-H)^−^ at *m*/*z* 593.083. The MS/MS spectrum showed product ions at *m*/*z* 473.108 (M-H-120)^−^ and at *m*/*z* 353 (M-H-210)^−^ resulting from sugar fragmentations. This compound was identified as apigenin-6,8-C-dihexose (vicenin-2) [6]. Compound **15** gave a precursor ion (M-H)^−^ at *m*/*z* 577.156. The product ions observed in the MS/MS spectrum are due to the sugar fragmentations. This compound was thus identified as chrysin-6,8-C-diglucoside [69, 70]. This is the first time that this flavonoid is reported in *M. oleifera*.

**Figure 8:**
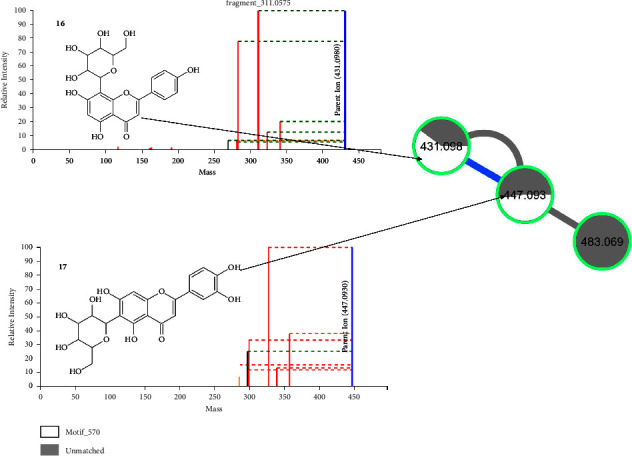
Fragmentation spectra of apigenin-8-C-hexose (**16**) and luteolin-8-C-hexose (**17**) in *M. oleifera* as annotated by MS2LDA.

Figure 8 shows the fragmentation spectra of an apigenin-related flavonoid and a luteolin-related flavonoid as annotated by motif_570 mass2motifs on MS2LDA approach. Motif_570 is characterized by fragment ions at *m*/*z* 575, 357, 341, 339, 327, 323, 311, 299, 283, 215, and 197 and neutral losses of 18, 36, 90, 108, 120, 148, and 162 amu. Compound **16** gave a precursor ion (M-H)^−^ at *m*/*z* 431.098. It gave a base peak fragmentation ion at *m*/*z* 311.058. Further fragments were observed at *m*/*z* 341.068, at *m*/*z* 323.053 due to the loss of H_2_O, and at *m*/*z* 283.063 due to the loss of a CO moiety. This compound was thus identified as apigenin-8-C-hexose (vitexin) [71]. Compound **17** gave a precursor ion (M-H)^−^ at *m*/*z* 447.093. The fragment ion observed at *m*/*z* 285.043 (M-H-162)^−^ was due to the fragmentation of the hexose sugar. This compound was thus identified as luteolin-8-C-hexose (orientin) [72].

### 3.6. Glycoisomerization of Flavonoids

*Moringa oleifera* has been reported to undergo glycosylation patterns in order to diversify its flavonoids. *Moringa oleifera* attaches different types of sugars to its flavonoid aglycones [73]. For example, quercetin is observed to attach different types of sugars to its aglycone structure, as observed in Figure 3. Furthermore, the glycosylation of flavonoids can undergo further chemical modification such as isomerization, acetylation, malonylation, and acylation. These modifications, however, bring about an analytical challenge because of the isomers are identified as structural artefacts. Some of the flavonoids undergo glycosylation through disaccharide sugar attachments [74]. Coelution of different flavonoids is often encountered in LC, which makes it difficult to characterize the flavonoid composition. However, MS has a high sensitivity by making use of multiple reaction monitoring (MRM) which helps to improve the selectivity of the flavonoids [75].

Compounds that have similar molecular formulae but different chemical arrangements are considered to be isomeric. For example, compounds kaempferol acetyl hexose (*m*/*z* 489), quercetin malonyl hexose (*m*/*z* 549), and isorhamnetin hydroxymethylglutaroyl hexose (*m*/*z* 621) with molecular formulae C_23_H_22_O_11_, C_23_H_22_O_11_, and C_28_H_28_O_11_, respectively, are considered to be isomeric (Table 2). These isomers have a similar molecular formula and molecular mass and are also observed to have similar fragmentation patterns. However, the chemical arrangement of these compounds differs, which could be due to a slight shift in the position of the glycosidic bond between the organic acid and the sugar that is conjugated to the aglycone structure as suggested by the authors in [26]. It, however, still remains a challenge to distinguish these molecules. There is, therefore, a need to develop advanced analytical techniques so as to be able to distinguish between molecules of such a nature.

Isobaric molecules were also observed in this study. Isobaric molecules are molecules with the same mass but are of different compound composition. In this study, isobaric flavonoids were observed to have similar precursor ion mass at *m*/*z* 609 and 447 and molecular formula C_27_H_30_O_16_ and C_21_H_20_O_11_, respectively. However, the compound composition differs. This observation thus makes these compounds isobaric. The flavonoids with molecular formula C_27_H_30_O_16_ and precursor ion mass *m*/*z* 609 were identified as quercetin rutinoside and kaempferol diglucoside, and those with molecular formula C_21_H_20_O_11_ and precursor ion mass *m*/*z* 447 were identified as kaempferol hexose and luteolin-8-C-hexose (orientin). These compounds were difficult to tell them apart using only an LC-MS spectrum. However, upon the untargeted LC-MS/MS approach for metabolite profiling, the difference in the fragmentation spectra was useful in the identification of these flavonoids and was thus easy to distinguish them, as can be seen in Table 3 [76].

## 4. Conclusions

The use of computational tools such as molecular networking highlighted the different molecular families that are found within *M. oleifera* and thus bringing insight into the chemical space of the plant. Unsupervised substructure annotation (MS2LDA) was useful in the annotation of Mass2Motifs of some of the flavonoids found within *M. oleifera*. An enhanced molecular network unraveled the different chemical classes found in this plant and thus revealed the metabolome of *M. oleifera*. Seventeen flavonoids (flavonols and flavones) were successfully annotated by MS2LDA in this study and confirm what has been previously reported in the literature. MS2LDA was also useful in the annotation of chrysin-6,8-C-diglucoside which is reported in *MO* leaves for the first time through this study.

In the existing literature, it has been documented that flavonoids in *M. oleifera* undergo glycosylation using various sugars as a mechanism to expand their chemical diversity. This glycosylation process has led to the detection of isomeric and isobaric flavonoids in our current study. The untargeted LC-MS/MS approach in combination with computational metabolomics tools such as molecular networking proved valuable in identifying isobaric molecules due to their distinct fragmentation patterns, thereby successfully accomplishing their identification. However, a challenge persists when it comes to identify isomeric flavonoids, primarily because traditional MS techniques struggle to differentiate them effectively. Consequently, the future application of alternative MS analyzers, such as orbitraps and ion mobility, will become essential in addressing this challenge, especially when hyphenated to other computational metabolomic tools such as a feature-based molecular networking.

## Figures and Tables

**Table 1:** Identification of flavonoids by UHPLC-qTOF-MS and their Mass2Motifs.

No	Molecular formula	*m*/*z* value	Fragment ions	Compound name
1	C_27_H_30_O_16_	609.197	343.048; 301.038; 300.028; 271.098; 255.028; 178.998; 151.003	Quercetin rutinoside
2	C_23_H_21_O_13_	505.098	301.038; 300.033; 271.023; 255.028; 178.998; 151.013	Quercetin acetyl hexose
3	C_23_H_22_O_11_	549.089	505.103; 463.088; 301.038; 300.033; 271.023; 255.028	Quercetin malonyl hexose
4	C_12_H_20_O_12_	463.087	301.038; 300.028; 271.033; 255.028; 178.998; 151.003	Quercetin hexose
5	C_24_H_22_O_14_	533.093	489.108; 285.043; 284.033; 257.048; 255.028; 229.053; 227.033	Kaempferol malonyl hexose
6	C_26_H_28_O_16_	592.785	489.108; 447.098; 285.043; 284.033; 255.028	Kaempferol rutinoside
7	C_27_H_30_O_16_	609.146	489.103; 447.098; 446.088; 327.048; 285.043; 283.023; 255.028	Kaempferol diglucoside
8	C_21_H_20_O_11_	447.093	285.043; 284.033; 256.038; 255.028; 227.033	Kaempferol hexose
9	C_23_H_22_O_11_	489.114	285.043; 284.033; 255.023; 227.033	Kaempferol acetyl hexose
10	C_27_H_30_O_16_	623.161	315.053; 314.043; 300.023	Isorhamnetin rutinoside
11	C_28_H_28_O_11_	621.146	559.148; 519.118; 477.103; 315.058; 314.043	Isorhamnetin hydroxymethylglutaroyl hexose
12	C_22_H_22_O_11_	477.079	315.058; 314.043; 299.018; 285.043; 271.023; 257.048; 243.028	Isorhamnetin hexose
13	C_24_H_24_O_13_	519.114	357.058; 315.048; 314.043; 299.018; 285.043; 271.023; 257.048; 243.028	Isorhamnetin acetyl hexose
14	C_27_H_30_O_15_	593.083	503.118; 473.108; 383.078; 353.068; 311.058	Apigenin-6,8-C-dihexose
15	C_27_H_30_O_14_	577.156	487.123; 457.118; 439.103; 397.093; 379.083; 367.083; 337.073; 309.078; 281.083	Chrysin-6,8-C-diglucoside
16	C_21_H_20_O_10_	431.098	341.068; 323.053; 311.058; 283.063; 281.048; 269.048	Vitexin
17	C_21_H_20_O_11_	447.093	357.063; 339.048; 327.048; 299.053; 297.043; 285.043	Luteolin-8-C-hexose

**Table 2:** Isomeric flavonoids identified in *Moringa oleifera* methanolic leaf extracts.

(M-H)^−^ (*m*/z)	Molecular formula	MS/MS fragmentation	Compound name
489.297	C_23_H_22_O_11_	559.148; 519.118; 477.103; 315.053; 314.043	Kaempferol acetyl hexose (isomer 1)
489.114	C_23_H_22_O_11_	559.148; 519.118; 477.103; 315.058; 314.043	Kaempferol acetyl hexose (isomer 2)
549.088	C_23_H_22_O_11_	505.103; 463.088; 301.038; 300.033; 271.023; 255.028	Quercetin malonyl hexose (isomer 1)
549.088	C_23_H_22_O_11_	505.098; 301.038; 300.033; 271.023	Quercetin malonyl hexose (isomer 2)
621.146	C_28_H_28_O_11_	559.148; 519.118; 477.103; 315.053; 314.043	Isorhamnetin hydroxymethylglutaroyl hexose (isomer 1)
621.146	C_28_H_28_O_11_	559.148; 519.118; 477.103; 315.053; 314.043	Isorhamnetin hydroxymethylglutaroyl hexose (isomer 2)

**Table 3:** Isobaric flavonoids identified in *Moringa oleifera* methanolic leaf extracts.

(M-H)^−^ (*m*/*z*)	Molecular formula	MS/MS fragmentation	Flavonoid name
609.197	C_27_H_30_O_16_	343.048; 301.038; 300.028; 271.098; 255.028; 178.998; 151.003	Quercetin rutinoside
609.146	C_27_H_30_O_16_	489.103; 447.098; 446.088; 327.048; 285.043; 283.023; 255.028	Kaempferol diglucoside
447.093	C_21_H_20_O_11_	285.043; 284.033; 256.038; 255.028; 227.033	Kaempferol hexose
447.093	C_21_H_20_O_11_	357.063; 339.048; 327.048; 299.053; 297.043; 285.043	Luteolin-8-C-hexose

## Data Availability

The data used to support the findings of this study are available on request from the corresponding author. Here are the links to some of the data herein: Molecular network-https://gnps.ucsd.edu/ProteoSAFe/status.jsp?task=2e87085876784b468ef6bceed356faf1MS2LDA-https://gnps.ucsd.edu/ProteoSAFe/status.jsp?task=fe71bab7fd1d4e63abafa885ba4f5013DEREPLICATOR-https://gnps.ucsd.edu/ProteoSAFe/status.jsp?task=83f72cba82de4ea1a00249fbbb7513adNAP-https://gnps.ucsd.edu/ProteoSAFe/status.jsp?task=7f37b53ad6d14c6a8446e8a78f970e08MolNetEnhancer-https://gnps.ucsd.edu/ProteoSAFe/status.jsp?task=a0fdc30e35ed4cf5b344a02c9ee03c76.
